# A microfluidic chip enables fast analysis of water microplastics by optical spectroscopy

**DOI:** 10.1038/s41598-021-89960-4

**Published:** 2021-05-18

**Authors:** Ahmed A. Elsayed, Mazen Erfan, Yasser M. Sabry, Rachid Dris, Johnny Gaspéri, Jean-Sébastien Barbier, Frédéric Marty, Fatima Bouanis, Shaobo Luo, Binh T. T. Nguyen, Ai-Qun Liu, Bruno Tassin, Tarik Bourouina

**Affiliations:** 1 ESYCOM, CNRS UMR 9007, Univ. Gustave Eiffel, ESIEE Paris, 93162 Noisy-le-Grand, France; 2 ECE Department, Faculty of Engineering, Ain Shams University, 1 El-Sarayat St, Cairo, 11517 Egypt; 3 LEESU, ENPC UPEC, 77455 Marne-la-Vallee cedex, France; 4 GERS-LEE Université Gustave Eiffel, IFSTTAR, 44344 Bouguenais, France; 5 COSYS-LISIS, Univ Gustave Eiffel, IFSTTAR, 77454 Marne-la-Vallée, France; 6 Laboratory of Physics of Interfaces and Thin Films, UMR 7647 CNRS/ Ecole Polytechnique, 91128 IPParis, Palaiseau France; 7 School of Electrical and Electronic Engineering, Nanyang Technological University, Singapore, 639798 Singapore

**Keywords:** Environmental monitoring, Hydrology, Chemical engineering, Techniques and instrumentation

## Abstract

Microplastics contaminating drinking water is a growing issue that has been the focus of a few recent studies, where a major bottleneck is the time-consuming analysis. In this work, a micro-optofluidic platform is proposed for fast quantification of microplastic particles, the identification of their chemical nature and size, especially in the 1–100 µm size range. Micro-reservoirs ahead of micro-filters are designed to accumulate all trapped solid particles in an ultra-compact area, which enables fast imaging and optical spectroscopy to determine the plastic nature and type. Furthermore, passive size sorting is implemented for splitting the particles according to their size range in different reservoirs. Besides, flow cytometry is used as a reference method for retrieving the size distribution of samples, where chemical nature information is lost. The proof of concept of the micro-optofluidic platform is validated using model samples where standard plastic particles of different size and chemical nature are mixed.

## Introduction

The contamination of the environment and marine water with microplastics is a growing issue that has been addressed by several studies for years now^1–10^. But most recently, the studies extended to address drinking water as well, revealing the fact that both tap and especially bottled water are contaminated with microplastics of different shapes, amounts, sizes and plastic types^11–20^. This led the World Health Organization (WHO) to issue reports focusing on drinking water quality and its impact on the human health^21,22^.

Microplastics refer to plastic particles of sizes less than 5 mm, and while a lower size limit is not strictly defined, it is commonly considered to be from 25 µm down to 1 µm^15^ depending on the limit of detection of analytical tools. The microplastic particles can be of different typologies, with different sizes, colors, and shapes such as fibers, spheres or fragments. The abundance of each typology is examined in some studies where water is analyzed for microplastics before and after passing through water treatment plants. In that case it was noticed that fragments within a detection limit of 20 µm range are most abundant in both untreated and treated water, followed by fibers then spheres^15^. This motivated several studies focusing on quantifying the microplastic particles in the different size ranges.

Concerning tap water, some reports discuss the analysis of particles having sizes > 100 µm^12,14^. Kosuth et al.^12^ collected samples from 14 different countries and their analysis revealed an average of 5.45 particles per liter, given that these particles were mostly fibers. Other studies confirmed that smaller particles (in the range of 1–100 µm) are much more abundant^11,13,15,20^, where treated tap water from three different water treatment plants were analyzed, showing an average count of particles of 443 ± 10, 338 ± 76 and 628 ± 28 per liter of treated water for the three different plants, respectively^15^.

Concerning bottled water, similar trends were noticed regarding the abundance of the smallest microplastics^13,16,18^. However, due to the lack of a standard procedure, the average amount of microplastics detected can vary significantly from one study to another^13,16^. For example, one study shows that the average number of particles > 100 µm is 10.4 particles per liter, and particles < 100 µm have an average of 325 particles per liter^16^. While another study shows that particles < 100 µm are much more abundant in the samples reaching 4 889 ± 5 432 particles per liter for reusable Polyethylene Terephthalate (PET) bottles, and 6 292 ± 10 521 particles per liter for glass bottles^13^.

This abundance in this dimension range led to an underestimation of the amounts of microplastic particles in earlier studies which focused on larger particles only, so more attention should be given to this size range.

The plastic types found in the recent studies include Polyester, Polyethylene Terephthalate (PET), Polyethylene (PE), Polypropylene (PP), Polyamide (PA) and others, but the abundance of each plastic type was shown to vary from one study to another, which can be related to different samples and brands analyzed in each study. For example, one study shows that PET is the most abundant type (with 57% contribution)^11^, while another study shows that PP is the most abundant (54%)^16^. Determining the plastic type of the contaminating particles is a key aspect in the analysis, as it helps determine the source of these particles and hence enables taking the necessary measures to limit water contamination.

**Figure 1:**
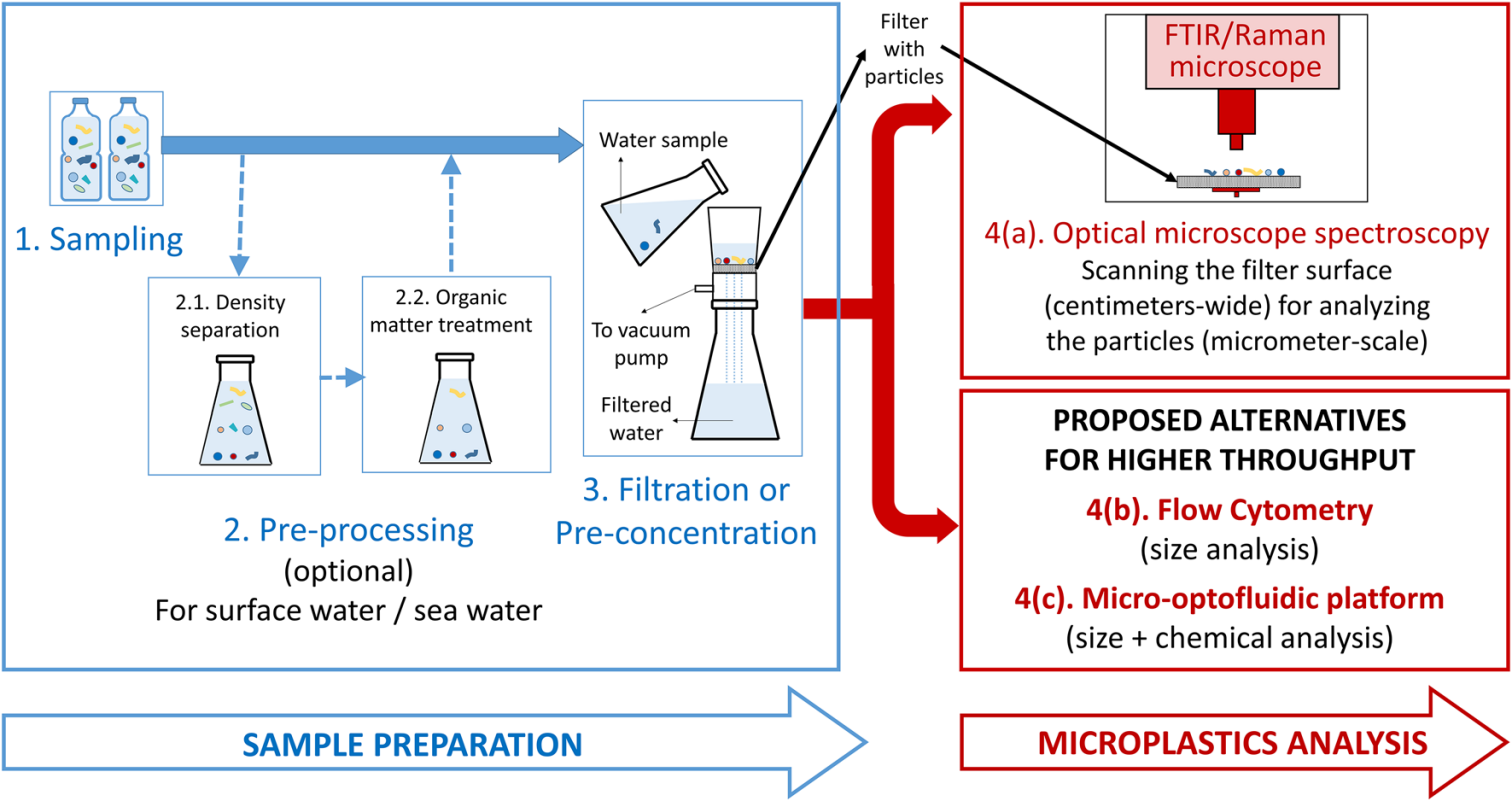
An illustration for water analysis process steps. First, common steps are required which include sampling (of tap water, bottled-water…etc.) Then, for some samples types (which can be more contaminated such as surface water) a step of pre-processing is required, where density separation is used in addition to treatment of organic matter to digest organic matter leaving only plastics in the sample. This is followed by filtration or pre-concentration of the water samples, then the analysis is done using either conventional methods of scanning the entire filters using an FTIR/Raman microscope, or using the proposed approaches for high-throughput analysis using flow cytometry or the platform utilizing small-sized microfluidic chips, which trap the particles in tiny dedicated reservoirs enabling faster spectroscopic analysis.

To date, all studies targeting the detection of microplastics in drinking water rely on time-consuming conventional techniques, which are illustrated in steps 1 to 4(a) in Fig. 1. The process starts in step 1 by sampling (of tap water, bottled-water, surface water, etc.…). Then a step of pre-processing (step 2) may be required for the most contaminated types (surface water, sea water for instance). In this case, density separation is used in addition to chemical treatment to digest organic matter leaving only plastics in the sample. The sample is then filtered (step 3) using small-pore filters, which can trap particles larger than the pore-size, in addition to some smaller particles that may adhere to the filter or other larger particles. The filters have a pore-size that varied for the different studies but ranged from 0.2 µm to few microns^11–13,15,17^. The resulting filters containing the accumulated particles can be analyzed using a number of methods. One method (not sketched in Fig. 1) includes the use of dye staining and microscope inspection. In this method, the water sample is treated with a fluorescence dye (such as Nile Red) that adsorbs to microplastics before filtering the water sample^2,16^. This dye fluoresces when illuminated using a proper light source (such as a high-powered blue LED) which helps identifying plastic particles as the dye mostly adsorbs to plastic particles only. However, the exact type of plastic cannot be determined in this case, in addition to other disadvantages that are discussed later.

For more accurate analysis of the microplastics and to determine the type of each plastic, a Raman or FTIR microscope can be used^11,13,16,23^ (step 4(a) in Fig. 1). In this case, a first option is that each particle is localized – either by visual inspection or by image analysis; the corresponding measured spectrum is compared with a database of plastic spectra. A second option is to make spectral imaging of the whole filter surface. Since the FTIR microscope uses a wide-band IR source, due to diffraction limits, there is a lower limit on the detectable particle size, which is in the order of 10 µm for FPA-based µFTIR microscopes, and a few micrometers for ATR-enabled FTIR microscopes, while for the Raman microscope this limit decreases to slightly below 1 µm, since visible light is used ^13,23^. Some techniques provide nano-scale analysis, such as nano-FTIR which can achieve a spatial resolution of 20 nm^24^, and nano-Raman imaging which demonstrates a spatial resolution of 100 nm^25^, but these techniques are not suitable for scanning relatively large surface areas, and hence are not considered in this work.

Using either an FTIR microscope or a Raman microscope to scan a whole filter manually can be a tedious and labor intensive process that can take tens of hours, in addition to the high cost and technical skills required for using these microscopes, which justify recent efforts towards full automation^26^.

In this paper, two alternative approaches for microplastic analysis are proposed to achieve high throughput and overcome the aforementioned disadvantages: (i) flow cytometry and (ii) a micro-optofluidic platform based on microfluidic chips.

Flow cytometry is an advanced technology used primarily for obtaining information about cells in a number of biological applications^27^. However, the capabilities of flow cytometers can be useful for other emerging applications such as the one presented in this work, where the counts, sizes and shapes of the particles can be obtained. Some experiments adopting this technique using standard particles are presented in the results section. The main drawback of this technique is that it does not enable determining the chemical nature of the particles, rather detecting only physical and morphological properties. This is addressed in the second approach of using a micro-optofluidic platform based on microfluidic chips for microplastic analysis which is presented next.

## Results

### Principle of chip-scale sorting, concentration and spectroscopic analysis

One motivation of our work is the abundance of the smallest microplastic particles (sized 1–100 µm), which led us to target specifically this class of particles and propose an efficient methodology, not only for their quantification in a timely manner, but also for their identification to recognize if the particle is plastic or not, and also to determine which type of plastic it is. We aim to achieve both tasks with high throughput, so as to drastically reduce analysis time, which is a key factor for monitoring and for enabling further large scale studies on microplastics in water. It should be noted that the maximum size of the analyzed particles using the proposed technique is not limited to 100 µm, but it can be extended to larger particles in the order of few hundred microns, and even up to 1 mm.

**Figure 2:**
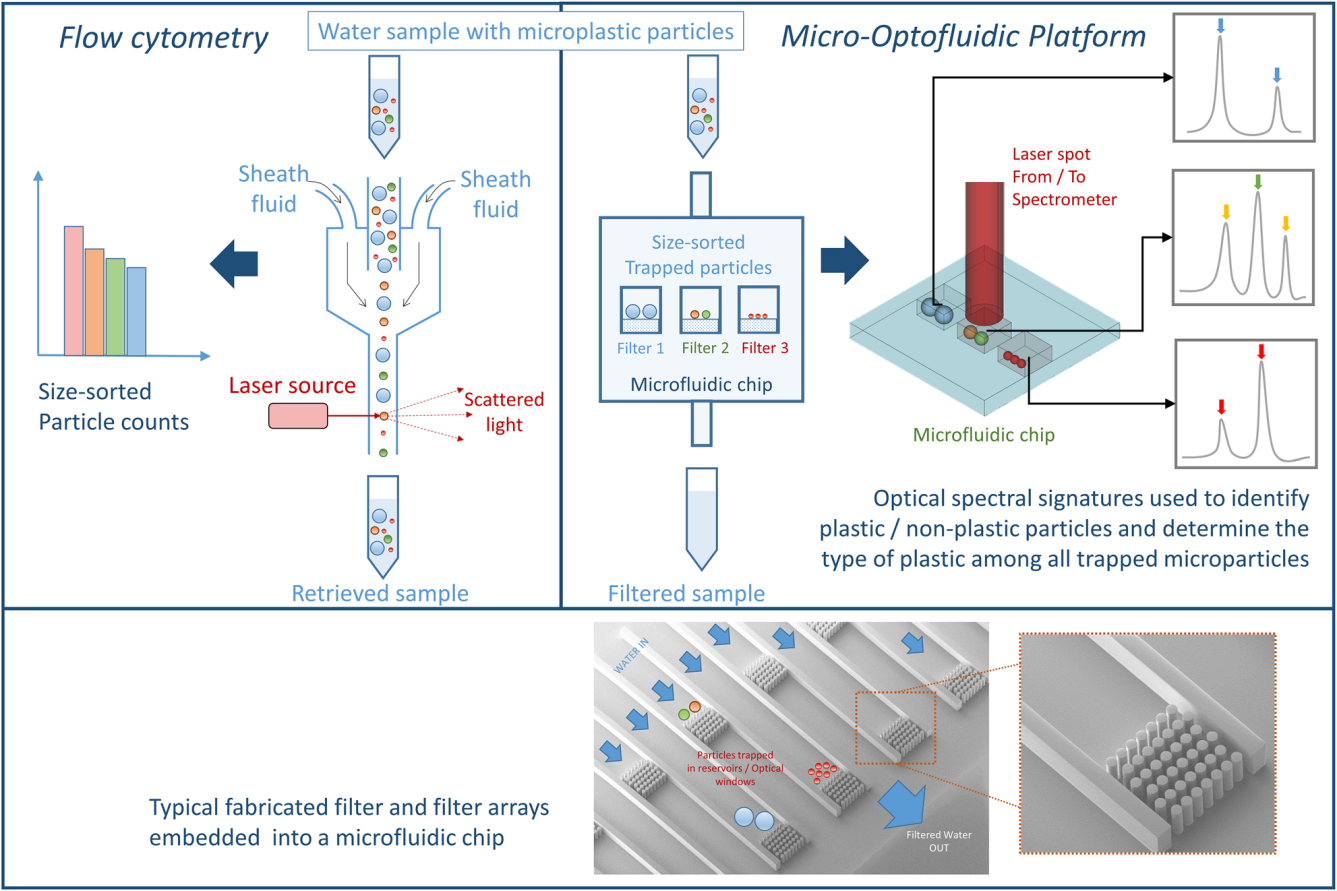
An illustration for the proposed analysis platform. On the left, flow cytometry is demonstrated which can be used as a reference technique to count and obtain statistics about the microparticles sizes and shapes, but the chemical nature information is lost. On the right, the proposed analysis platform is demonstrated which involves a microfluidic chip used to sort and trap the microparticles, in addition to enabling imaging and the identification of the chemical nature of the trapped particles using spectroscopic techniques. The particles are trapped in a series of reservoirs each terminated with a filter consisting of an array of pillars.

The proposed micro-optofluidic platform includes a microfluidic chip developed to achieve numerous functions, where it can sort the particles according to their size, and trap them in dedicated ultra-compact reservoirs on-chip. This enables imaging and spectroscopic analysis of these particles in a time-efficient manner. An illustration for the proposed analysis platform is shown in Fig. 2, along with flow cytometry which can serve as an excellent reference technique for particle size distribution.

The chips are tested using model plastic particles, which size and nature characteristics are known a priori, and which have been diluted in an ultra-pure water solution under controlled concentrations. As a proof-of-concept, several demonstrations were conducted where the particles of different sizes are sorted and trapped in different dedicated reservoirs, then imaged and analyzed using a number of spectroscopic techniques including a Raman spectrometer, a Raman microscope, and an FTIR microscope; the results of those techniques are eventually compared. The chip design includes reservoirs that range from about 100–300 µm in lateral dimensions, enabling concentration of all trapped particles in such a very tiny sub-millimeter space –compared to the centimeter-scale filters, which is a key requirement for further fast spectroscopic analysis of the sorted particles. Also, the chip reservoirs can be designed to accommodate particles of different size ranges. These ultra-compact reservoirs are seen as a huge advantage compared with the conventional techniques that use centimeter-scale filters, for which scanning the entire surface is extensively time-consuming. Hence, this technique is designed to be time-efficient and low cost thanks to the advantages of microfluidics, accurate and more practical thanks to the easiness of coupling optical beams to implement spectroscopic analysis.

### Flow cytometry measurements

Flow cytometers can obtain accurate data about particle counts, shapes and sizes in addition to other chemical and physical properties. Also, some high-end flow cytometers are equipped with a light source and high speed cameras that can obtain up to thousands of images per second, which can provide additional essential information about the particles in the analyzed sample.

**Figure 3:**
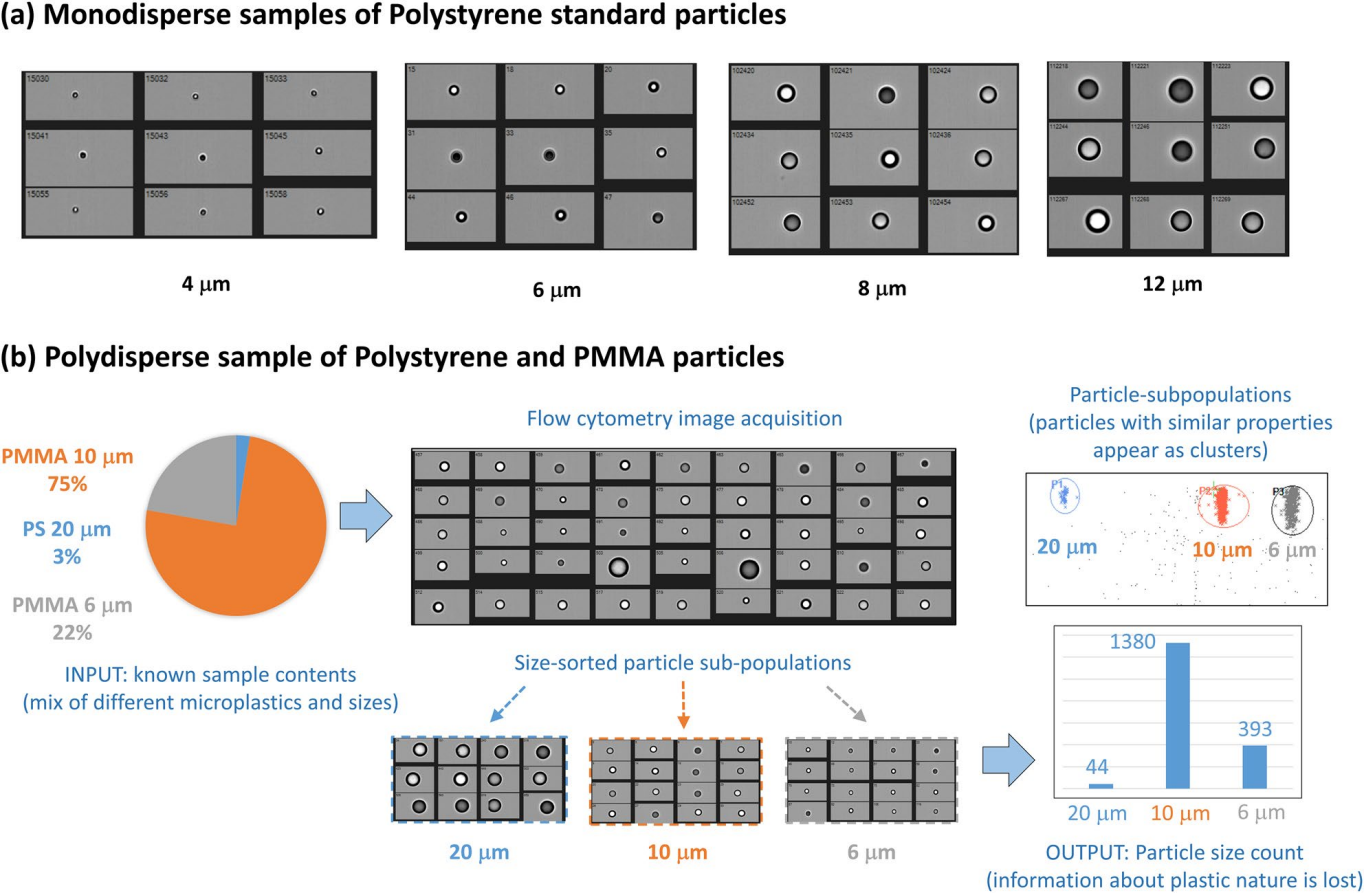
Flow cytometry results. Model solutions of ultra-pure water and standard microplastic particles of different types and sizes are analyzed using the flow cytometer. (**a**) Obtained images for individual particles in different monodisperse samples of standard PS spheres of different sizes (4 µm, 6 µm, 8 µm and 12 µm) (**b**) A polydisperse sample of (PMMA 6 µm, PMMA 10 µm, PS 20 µm) is analyzed and images for each particle in the solution is obtained, along with statistics about the counts of particles of different sizes in the sample.

This is validated with a set of experiments using the flow cytometer (specified in the Methods section), where standard plastic particles of known sizes are introduced into a small volume of ultra-pure de-ionized water and are analyzed using the flow cytometer. First, monodisperse samples (each sample containing particles of one size) are tested. These samples include standard Polystyrene (PS) spheres having sizes of 4 µm, 6 µm, 8 µm and 12 µm. Bright-field images for individual particles in the different samples are obtained, and the results are shown in Fig. 3(a). These images can provide useful information regarding particle shapes and sizes. The particle sizes can be calculated from the obtained images using machine-learning techniques developed for this application^28^.

Next, we considered a polydisperse sample containing both Polystyrene spheres of size 20 µm, and PolyMethylMethAcrylate (PMMA) spheres of sizes 10 µm and 6 µm. Images for individual particles could be obtained successfully, and classification of particles of different sizes into populations, is achieved thanks to image analysis. This classification process is flexible and can accommodate particles of larger sizes, up to a few hundred microns. Also, other parameters such as the aspect ratio of the particles can be used to classify them into different clusters, allowing the classification of particles of different shapes including fibers. The obtained results are demonstrated in Fig. 3(b). The particles measured were clustered into three main populations, which correspond to the three different particles sizes prepared in the solution (20 µm, 10 µm and 6 µm). The counts of each particle size could be obtained and displayed, and images for each particle in the different population clusters could be obtained. Despite the infeasibility of identifying plastic types using this technique, it can still provide valuable information regarding the counts, shapes and sizes of the particles in water samples, whatever if they are plastic or not. It is worth mentioning that flow cytometry has been already applied for water analysis for the purpose of counting and classification of microparticles thanks to image processing and deep learning approaches^29^. Although this was efficient for living micro-organisms including cyanobacteria, diatoms, green algae and red algae according to their specific images that serve as signatures, it was much less efficient for recognizing microplastics, which do not have a repeatable image signature like living micro-organisms.

The size and cost of flow cytometers can force some limitations on the applications, hence recently many researchers have targeted integrating this technology on-chip to suit a wider range of applications^30–32^. One example includes implementing a refractive index cytometer on-chip, which uses a Fabry-Pérot cavity perpendicular to the flow channel and a laser source to illuminate the flowing cells. The shift in the resonance response is used to estimate the refractive index of the cell, and the asymmetry in the response curve can be used to distinguish different types of cells^30^. However, in our application using such a technique is not sufficient to determine the chemical nature of the microplastics, but this is addressed in more details in the future challenges section.

### Microplastic particles trapping and sorting on-chip

**Figure 4:**
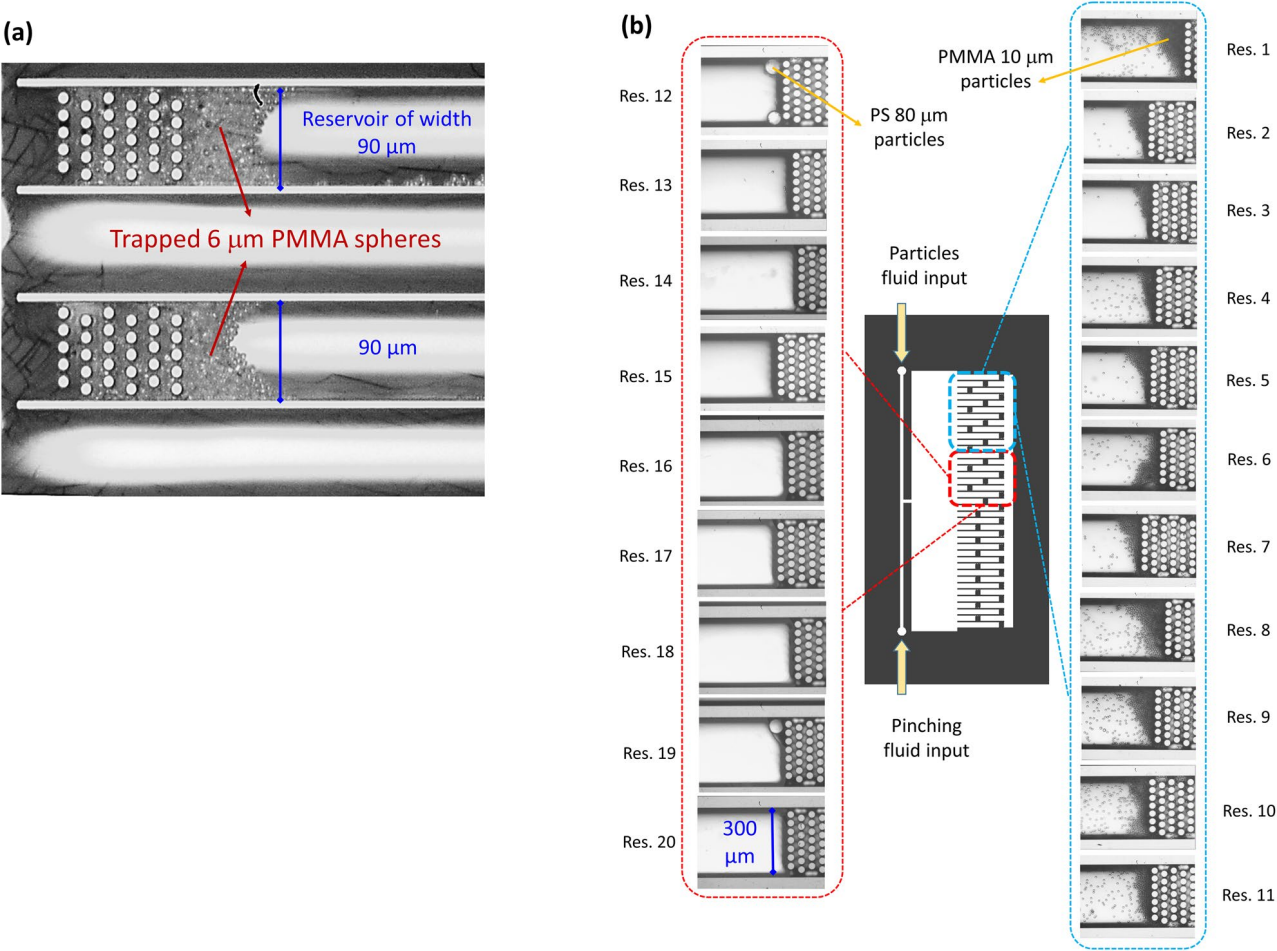
Particle trapping and sorting on-chip. (**a**) Trapping of monodisperse population of particles, consisting of standard PMMA particles of diameter 6 µm. (**b**) Sorting and trapping of polydisperse population (PMMA 10 µm and PS 80 µm standard particles) into multiple different reservoirs.

The proposed microfluidic chips can be used to achieve at least two functionalities, one is to trap the microplastic particles in dedicated reservoirs on-chip, and the other is to sort the particles according to their size in different reservoirs. The chips are tested using a solution of ultra-pure de-ionized water with standard spherical plastic particles using the setup shown in the supplementary material (Supplementary Fig. S1). The solution is inserted into the chips, and images for the reservoirs are obtained showing the trapped particles. First, some chips were used to trap a monodisperse population of PMMA 6 µm standard particles which are shown in Fig. 4(a).

Next, a polydisperse population of PMMA 10 µm and PS 80 µm particles is sorted using one of the proposed chips. The technique used for sorting the particles is called Pinched Flow Fractionation (PFF), and is discussed in more details in the Methods section and in the supplementary material (refer to Supplementary Fig. S2). In this case the larger particles (PS 80 µm diameter) are expected to accumulate in reservoirs near the chip center, while the smaller particles (PMMA 10 µm diameter) are expected to accumulate in reservoirs near the chip edge. The solution is inserted into the chip (using the setup shown in﻿ Supplementary Fig. S1) with a flow rate in the range of tens of micro-liters per minute. The analyzed volume of the concentrated sample is less than 1 mL. The sorting of particles according to their size was eventually confirmed after taking images on all the reservoirs, where it can be seen in Fig. 4(b) that the middle reservoirs near the chip center contain mainly the larger particles (PS of 80 µm diameter, found particularly in reservoirs 12 and 19), while the reservoirs near the chip edge contain the smaller particles (PMMA of 10 µm diameter, found in all reservoirs from reservoir 1 to 11).

### Microplastics identification and classification by spectroscopy on-chip

To determine the chemical nature of the microplastic particles trapped on-chip, both Raman and FTIR spectroscopy were implemented on the micro-reservoirs full of microparticles.

**Figure 5:**
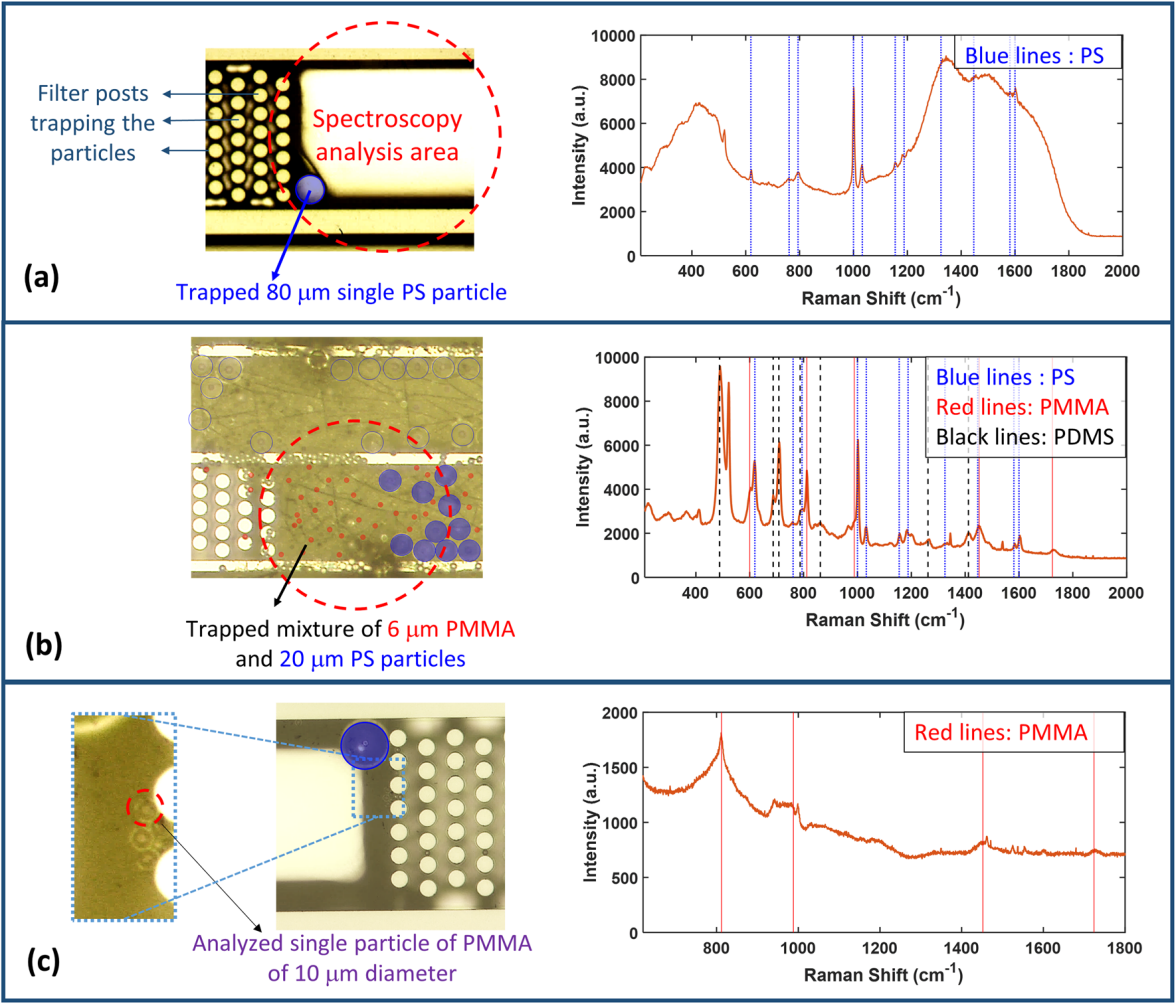
Raman spectrometer and Raman microscope measurements. (**a**) A microscope image of the chip reservoir containing a trapped PS particle of diameter 80 µm, along with the obtained spectrum for this particle using the Raman spectrometer. (**b**) A microscope image of the chip reservoir containing a mixture of 6 µm PMMA particles and 20 µm PS particles, along with the obtained spectrum for this mixture using the Raman spectrometer. (**c**) A microscope image of the chip reservoir containing a number of particles, where only one particle is selected and analyzed (PMMA 10 µm) using the Raman microscope with the help of the small-spot size of 1 µm. (**a**–**c**) Dashed-line red circles denote the area of the incident light spot for the analysis.

First set of experiments includes using a Raman spectrometer (specified in the Methods section) and the setup is shown in the supplementary material in Supplementary Fig. S3(a). First, a measurement for a single particle is presented where in this case the particle position is identified using a microscope and the target reservoir is determined. This reservoir contains a single 80 µm PS particle (a microscope image for the reservoir is shown in Fig. 5(a)), and is aligned with the laser spot, shown as a dashed-line circle, to analyze the particle. The obtained Raman spectrum is shown in Fig. 5(a) demonstrating excellent agreement between the measured Raman peaks and the theoretical peak positions shown as vertical blue lines^33^. It should be noted that this chip is top-sealed using glass (Borofloat 33), which causes the fluorescence signal observed in the spectrum between 1200–1800 cm^-1^ due to the presence of rare earth impurities.

Next, a measurement for a mixture of standard particles is presented consisting of PMMA 6 µm diameter and PS 20 µm diameter. A microscope image for the reservoirs containing these trapped particles is shown in Fig. 5(b)). This chip was sealed for microfluidic operation using a PDMS patch, so the Raman peaks of PMMA, PS and PDMS should be visible in the measured spectrum. The obtained Raman spectrum is shown in in Fig. 5(b). The expected positions of the Raman peaks for these different materials are shown as vertical lines where the black dashed lines represent PDMS, the red solid lines represent PMMA, and the blue dotted lines represent PS^33–35^. It can be noticed that an excellent match between the theoretical positions of the Raman peaks and the measured ones is achieved.

The second set of experiments includes using a Raman microscope for standard particles trapped on-chip. The details about the used Raman microscope are discussed in the Methods section. This device enables the analysis of smaller single particles where it uses a laser spot of size 1 µm, and targeting the smaller particles can be easily achieved with the help of live-imaging. The setup used is shown in the supplementary material in Supplementary Figure S3 (b). The presented measurement is for a single PMMA particle of diameter 10 µm -a microscope image is shown in Fig. 5(c). It should be noted that the intensity of the Raman peaks of PMMA is very weak compared to that of the Silicon substrate of the chip, so to emphasize the Raman peaks of PMMA only the Raman shift range of 620–1800 cm^-1^ was measured and is shown in this figure, demonstrating excellent agreement with the expected Raman peak positions^34^.

**Figure 6:**
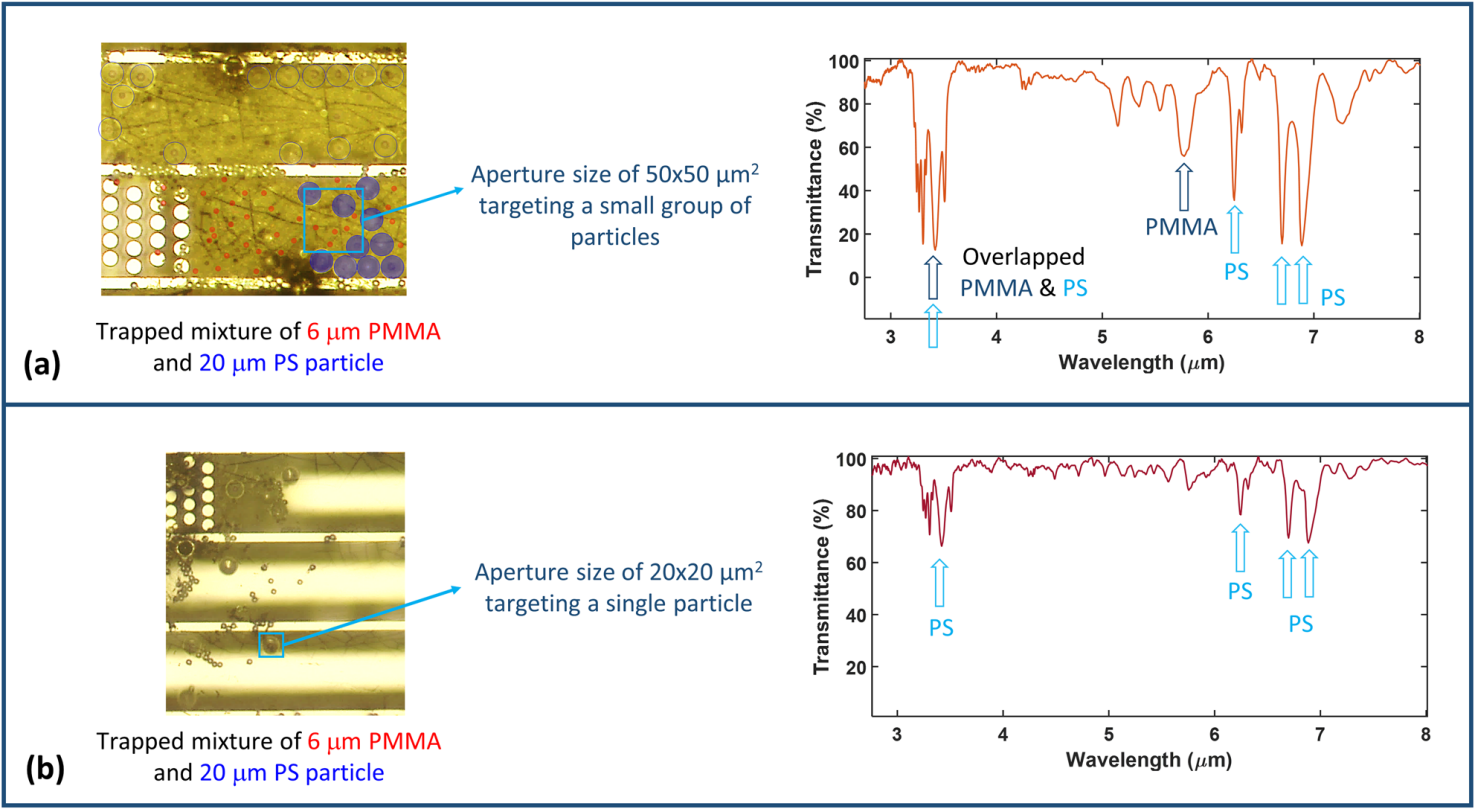
FTIR microscope measurements. (**a**) A microscope image of the chip reservoir containing the trapped particles of 6 µm PMMA and 20 µm PS, given that the aperture size is 50 × 50 µm^2^ and is highlighted in dashed blue. Shown next to it is the obtained IR spectrum for the trapped particles showing the absorption dips of both PMMA and PS. (**b**) A microscope image showing the analyzed single particle given that the aperture size is 20 × 20 µm^2^ and is highlighted in red, and shown next to it the obtained IR spectrum for the targeted particle showing the absorption dips of PS.

The third set of experiments was conducted using an FTIR microscope. The FTIR microscope uses an aperture of adjustable size, allowing the measurement of entire reservoirs or single particles. The details about the used FTIR microscope are discussed in the Methods section. First a mixture of standard particles of PMMA 6 µm diameter and PS 20 µm is analyzed (which is the same sample measured using the Raman spectrometer, and previously demonstrated in Fig. 5(b)). The aperture in this case was adjusted to a size of 50 × 50 µm^2^ targeting a group of particles in the middle of the reservoir (shown in Fig. 6(a)), and the obtained IR spectrum is shown in the same figure demonstrating the unique absorption dips of both PMMA and PS^36,37^.

Next, the aperture was reduced to 20 × 20 µm^2^ targeting a single PS particle of size 20 µm (shown in Fig. 6(b)), and the obtained spectrum is shown in the same figure demonstrating the unique absorption dips of PS^37^. It should be noted that the smaller aperture size leads to less optical power reaching the FTIR microscope detectors decreasing the obtained signal-to-noise ratio, hence affecting the quality of the obtained spectrum.

## Discussion

The demonstrated results serve as a proof-of-concept for the proposed micro-optofluidic analysis platform, where the microplastic particles were successfully trapped and sorted using microfluidic chips, then spectroscopically analyzed using several techniques in a time-efficient manner thanks to the designed chips. This is further discussed and compared to conventional techniques as follows.

Conventional techniques for detecting microplastics can include a step of dye staining (such as Nile Red) that adsorbs to plastics then the fluorescence of this dye is observed^2,16^. This can be used for rapid estimation of the amounts of microplastics, however the plastic type cannot be determined. Also, this method requires several steps for sample preparation in addition to being time-consuming where the staining dye has to be incubated for 30–60 min, then manual inspection of the filtered sample over the entire used filter is required, which can take hours for a standard 25.4 mm wide filter, and can also be inaccurate as the dye may adsorb to some non-plastic particles, and they can be mistaken for plastics. Hence, using a spectroscopic technique such as FTIR microscopy is still required for analyzing the suspected particles.

A more robust technique involves using an FTIR or Raman microscope to examine the filtered particles, however scanning an entire 25.4 mm filter using an FTIR microscope can take tens of hours^15,17,26^, while a Raman microscope will be orders of magnitudes slower due to the smaller spot size. To reduce this to a more feasible time, researchers scan a portion of the filter (10% of the filter area^17^, or 25% of the filter area for example^15^) and extrapolate the results, but this can be inaccurate as the microplastic particles are not guaranteed to be evenly distributed across the filter. Another method is to scan a number of random spots on the filter, for example five spots each of an area of 1 mm^2^ then extrapolate the results^13^.

The proposed platform based on microfluidic chips hence solves this issue by trapping the particles in dedicated reservoirs of limited size (the reservoirs are in the order of 100 µm to 300 µm wide), in addition to sorting the particles according to their size for some designs. It should be noted that a pre-concentrated sample is required to get a statistically representative sample for the analysis, where for example 1 L of bottled water is concentrated into a 1 mL sample by filtering the particles on standard filters, then re-suspending them into a small volume of ultrapure water before inserting it into the proposed microfluidic chips. Applying a spectroscopic technique such as FTIR microscopy in this case on a pre-concentrated sample reduces the analysis time significantly. This is demonstrated in Table 1 where it can be noticed that conventional techniques involving either manual or automated particle inspection with FTIR analysis can take tens of hours (12 to 15 h and about 38 h respectively)^26^, and at best a few hours using FTIR microscopes with larger (128 × 128 pixel) FPA^38^. While techniques such as flow cytometry require only about 15 min for a pre-concentrated sample of a volume 500 µL, and the designed microfluidic chips enable spectroscopic analysis in about 20 min and 14 min for FTIR-microscope scanning and Raman-spectrometer averaging respectively.

**Table 1:** A comparison of microplastics analysis time using different techniques.

Conventional techniques in the literature for analyzing microplastics^26,‡^		Analysis time for a pre-concentrated sample of 500 μL	Flow cytometry	Micro-optofluidic chip	
Manual inspection and FTIR analysis^26^	Automated analysis pipeline with focal plane array (FPA)^26^		~ 15 min	Fluid Injection^§^: 12 min	
720—900 min	2320 min			FTIR scanning*: < 7 min	Raman spectral averaging**: < 2 min
				Total < 20 min	Total < 14 min

^‡^ The study performed in this reference^26^ was on a marine sediment but the spectroscopic analysis process is identical and appropriate for drinking water samples after sample preparation steps.

^§^The microfluidic chip is first filled with the pre-concentrated sample of a 500 μL at a flow rate of nearly 42 μL/min, corresponding to the injection time is less than 12 min.

*For a typical chip whose unit reservoir size is 300 μm wide and 1 mm long, the total area of 20 reservoirs, each for different particle sizes, is 6 mm^2^, which is 2 orders of magnitude smaller than the area of a standard filter of diameter 25.4 mm, hence if the latter requires 10 h for scanning, then our set of micro-reservoirs can be scanned in less than 7 min.

**Assuming a relatively long averaging time for excellent signal (5 s per reservoir), and a 1 mm^2^ laser spot, then the total should be less than 2 min (20 reservoirs with 5 s each = 100 s or 1.67 min).

Another advantage of the proposed technique is the accumulation of smaller particles in a dedicated reservoir, where a better spectroscopic signal can be obtained for the collectively trapped particles. These particles can be too small individually (in the order of a few microns or even one micron) and can normally be missed, or have a very weak signal in case of using a Raman microscope, or can simply be unmeasurable in case of using an FTIR microscope.

Hence, the proposed micro-optofluidic platform offers a fast and cost-efficient solution for quantitative analysis and identification of microplastics in drinking water, enabling large-scale studies of water quality.

### Future challenges

A number of challenges exist in the design of the micro-opto-fluidic platform and in the related testing process. First, there is still a margin of progress in improving size-sorting efficiency. For instance, it can be noticed in Fig. 4(b) that the standard largest particles are fewer in numbers. Also, it is noticed that the particles are spread across a number of reservoirs, which can be related to the flow of the particles at an arbitrary position in the particles input channel. This issue can be tackled by using a technique such as flow-focusing to ensure that the particles are centered before entering the pinched segment, hence they are expected to accumulate in fewer reservoirs enabling the sorting of particles of multiple size ranges simultaneously using the same design.

The above-mentioned imperfections could be avoided by not considering size sorting at all. Alternatively, one can consider the implementation of flow-cytometry on-chip, where each flowing individual particle can be imaged using the appropriate camera, obtaining information about its shape, size and color, in addition to counting the particles flowing in the sample, which increases the analytic capabilities of the proposed platform even further.

The target of our future work is to test the proposed platform using real water samples, such as bottled-water and tap water samples. These samples will require initial steps of sampling and pre-concentration (using the setup shown in Supplementary Fig. S4) before the analysis using the proposed platform, then its performance can be compared with conventional techniques regarding the analysis time and practicality. It is expected that real water samples may contain non-plastic particles in addition to plastics, which should not affect the identification of the chemical nature of the particles due to the use of reliable spectroscopic techniques. Also, the accuracy of determining particle counts, shapes and sizes using future designs can be assessed by comparing the results with those of flow cytometry.

## Methods

### Chip design and fabrication

The chips are fabricated using MEMS technology on Silicon wafers using Deep Reactive Ion Etching (DRIE) technique. First, front-etching of the channels and different features of the designs are achieved using DRIE, then back-etching of the wafer is achieved to etch the through-holes needed for fluid inputs and outputs of the microfluidic chips. Finally, the chips are sealed from the top using either glass-bonding or using PolyDiMethylSiloxane (PDMS) which is adhered to the Silicon chips using oxygen plasma treatment. The fabrication process steps are listed in more detail in the supplementary material (Supplementary Fig. S5).

### Particle sorting

There are a number of well-known techniques for sorting microscale particles and biological cells which can adopted in this application, including Deterministic Lateral Displacement (DLD)^39^, Field Flow Fractionation (FFF)^40^, and Pinched Flow Fractionation (PFF)^41^ which is the one used in this work. In this technique two inlets are required, where one is used to input the fluid containing the particles, and the other inlet is used to input a pinching fluid at a higher flow rate. Then the two fluids flow through a segment of a smaller width named the pinched segment, which causes the particles in the first fluid to be pushed against the channel wall, and since the particles have different sizes then the positions of their centers of mass will cause each size to follow a different streamline, leading to their separation in the broadened segment^41^. This technique is further demonstrated in the supplementary material (Supplementary Fig. S2). After spatially separating the particles of different sizes they are then trapped in dedicated reservoirs on-chip. The reservoirs are terminated with a filter that allows the flow of water out of the chip but traps the particles to allow further analysis.

### Microfluidic operation setup

The setup includes a dual syringe pump that is used to precisely control the flow rates of the water sample and the pinching fluid (required for the PFF sorting technique). The syringe pump used is Kd-Scientific KDS 210 Legacy dual syringe pump. This pump pushes the two syringes with the same speed, so to achieve the desired ratio between the water sample flow rate and that of the pinching fluid the syringe volumes have to be different, and are selected to achieve the required flow rate ratio of about 1:6. To fix and connect the microfluidic chip to the input and output tubes a manifold is used, which is made of PTFE (fabricated by ProtoLabs, UK). The input and output tubes are made of Teflon (supplied by Darwin Microfluidics) to ensure minimal adherence of plastic particles to their inner walls^42^. The setup is shown in the supplementary material (Supplementary Fig. S1).

### Standard microplastic beads

Synthetic microplastic beads (from Thermo Fisher Scientific, Duke Scientific, Polysciences Inc. and Microbeads AS) of different sizes (4 μm, 6 μm, 8 μm, 10 μm, 12 μm, and 20 μm) samples were spiked (separately for monodisperse samples, and with certain combinations for polydisperse samples) into 1 mL of de-ionized ultra-pure water. The materials of the beads include PolyMethylMethAcrylate (PMMA) and Polystyrene (PS).

### Raman spectrometer

The spectrometer used is OndaVia Raman Spectrometer utilizing a 785 nm laser excitation of adjustable power that can reach 60 mW, and it can detect Raman shifts for the range of 200–2000 cm^-1^ with a resolution of 4 cm^-1^. Attached to it an objective of 40 × magnification, with numerical aperture of 0.5, a working distance of 3 mm and a 1.1 mm thick glass cover. The chip is placed on a 2-axis positioner used to align the chip and the target reservoir under the laser spot.

### Raman microscope

The Raman microscope used is Horiba Jobin Yvon LabRam HR800 utilizing a 532 nm laser with a spot size of 1 µm, and a 10 × objective lens. The small spot size enables targeting small microplastic particles individually, where the spectrum of each particle can be obtained. The Raman microscope enables live-imaging where the trapped particles can be aligned with ease with the laser spot. The sample is illuminated using a white-source for imaging and is precisely moved to desired spots with the help of a motorized stage.

### FTIR microscope

The used FTIR microscope is ThermoFischer Nicolet iN10 utilizing a wide-band infrared light source and an adjustable aperture of a minimum size of 15 × 15 µm^2^. This limits the minimum size of the detectable microplastic particles, with a trade-off between the aperture size and the signal-to-noise ratio (SNR). However, it can be used to detect single particles of sizes greater than about 20 µm. The obtained spectrum can be compared to a database of plastic-spectra where the highest matching plastics are identified.

### Flow cytometer

The flow cytometer used in this work is Amnis ImageStream X MKII, which can detect up to 5000 events per second, with multiple detection channels including bright field imaging, fluorescence, and light scattering detection. The equipped camera can capture images of the flowing particles. The data obtained for the sample is analyzed using IDEAS Analysis software, which enables the classification of the analyzed particles and dividing them into different populations according to the desired parameters.

## Supplementary Information

Supplementary Information.
