# Supplementary material for: A microfluidic chip enables fast analysis of water microplastics by optical spectroscopy
Source: Sci Rep. 2021 May 18;11:10533. doi: 10.1038/s41598-021-89960-4 (PMC8131687; doi:10.1038/s41598-021-89960-4)
Supplement: Supplementary file 1 — Supplementary Information. [file 41598_2021_89960_MOESM1_ESM.docx]

# **Supplementary Information**

**A microfluidic chip enables fast analysis of water microplastics by optical spectroscopy**

Ahmed A. Elsayed^a^, Mazen Erfan^a,b^, Yasser M. Sabry^a,b^, Rachid Dris^c^, Johnny Gaspéri^c^, Jean-Sébastien Barbier^c^, Frédéric Marty^a^, Fatima Bouanis^d,e^, Shaobo Luo^a,f^, Binh T.T. Nguyen^f^, Ai-Qun Liu^f,a^, Bruno Tassin^c,^*, Tarik Bourouina^a,^*

^a^ *ESYCOM, CNRS UMR 9007, Univ. Gustave Eiffel, ESIEE Paris, F-93162 Noisy-le-Grand, France*

^b^ *Ain Shams University, Faculty of Engineering, ECE Department, 1 El-Sarayat St., 11517, Cairo, Egypt*

^c^ *LEESU, ENPC UPEC, F 77455 Marne-la-Vallee cedex, France*

^d^ *GERS-LEE Université Gustave Eiffel, IFSTTAR, F-44344 Bouguenais, France*

^e^ *COSYS-LISIS, Univ Gustave Eiffel, IFSTTAR, F-77454 Marne-la-Vallée, France*

^f^ *Laboratory of Physics of Interfaces and Thin Films, UMR 7647 CNRS/ Ecole Polytechnique, IPParis, 91128 Palaiseau-France.*

^g^ *School of Electrical and Electronic Engineering, Nanyang Technological University, Singapore 639798*

* Corresponding authors: [tarik.bourouina@esiee.fr](mailto:tarik.bourouina@esiee.fr) ; [Bruno.tassin@enpc.fr](mailto:Bruno.tassin@enpc.fr)

# Microfluidic operation and setup

The setup used for the chip operation is shown in Figure S1. It includes a dual syringe pump that is used to precisely control the flow rates of the water sample and the pinching fluid (required for the PFF sorting technique). The syringe pump pushes the two syringes with the same speed, so to achieve the desired ratio between the water sample flow rate and that of the pinching fluid the syringe volumes have to be different, and are selected carefully to achieve this flow rate ratio. To fix and connect the microfluidic chip to the input and output tubes a manifold is used, and the tubes used are made of Teflon to ensure minimal adherence of plastic particles to their inner walls ^S1^.


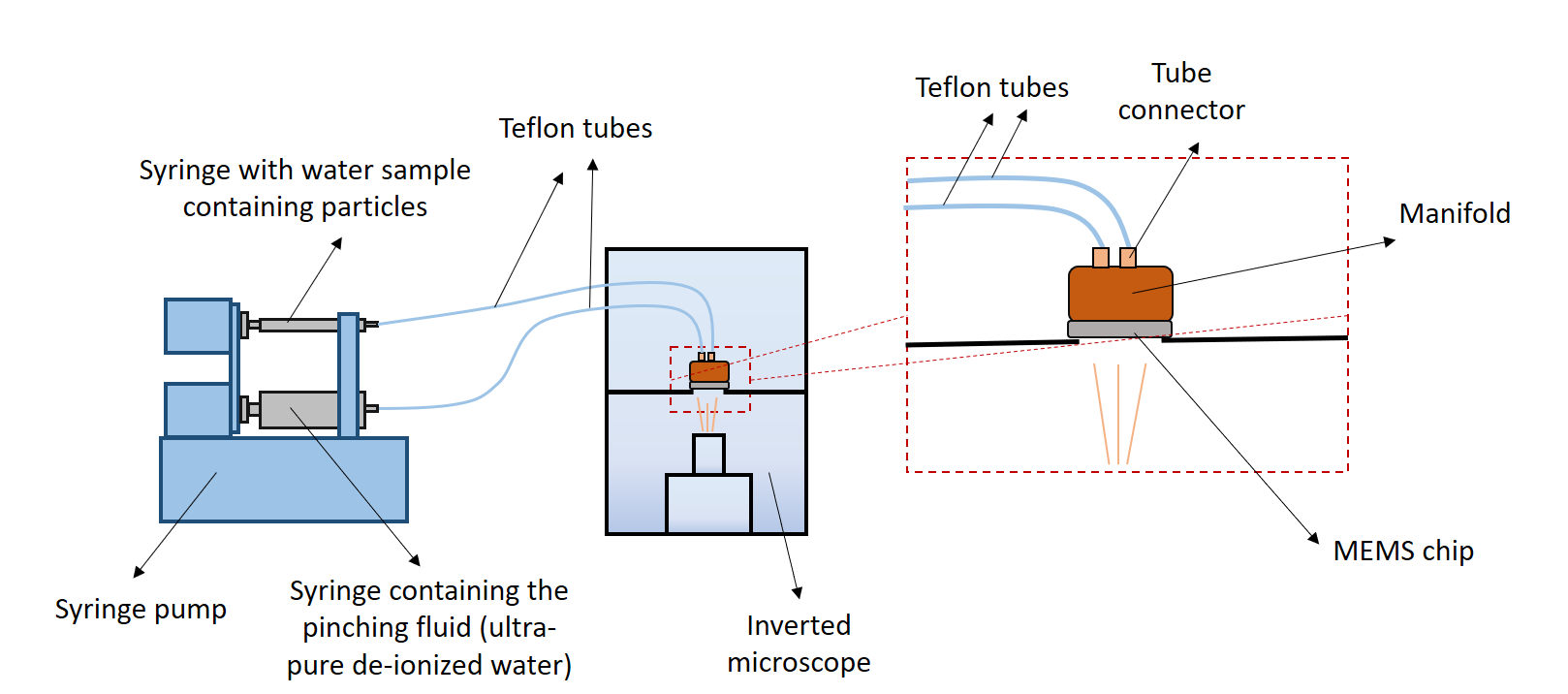


Figure S1: Setup used for operation of the microfluidic chip.

# Particle sorting – Pinched Flow Fractionation (PFF)

PFF is the chosen technique in this work to sort the microplastic particles and is presented briefly in this section. In this technique two inlets are required, where one is used to input the fluid containing the particles, and the other inlet is used to input a pinching fluid at a higher flow rate. Then the two fluids flow through a segment of a smaller width named the pinched segment, which causes the particles in the first fluid to be pushed against the channel wall, and since the particles have different sizes then the positions of their centers of mass will cause each size to follow a different streamline, leading to their separation in the broadened segment ^S2^.

The efficiency of spatial separation of the particles can be enhanced by decreasing the width of the pinched segment, and also by increasing the angle between the two inlets where maximum efficiency is achieved with an angle of 180^o^ as for the design shown in Figure S2. The typical ratio of the flow rates for the fluid with particles to the pinching fluid is 1:6 ^S2^. The position Y of a particle of diameter D_p_ in the broadened segment of width W_B_ (given that the pinched segment has a width W_P_) can be calculated using the following equation ^S2^:

$$Y=\left( W_{P}- \frac{D_{P}}{2} \right)\frac{W_{B}}{W_{P}}$$

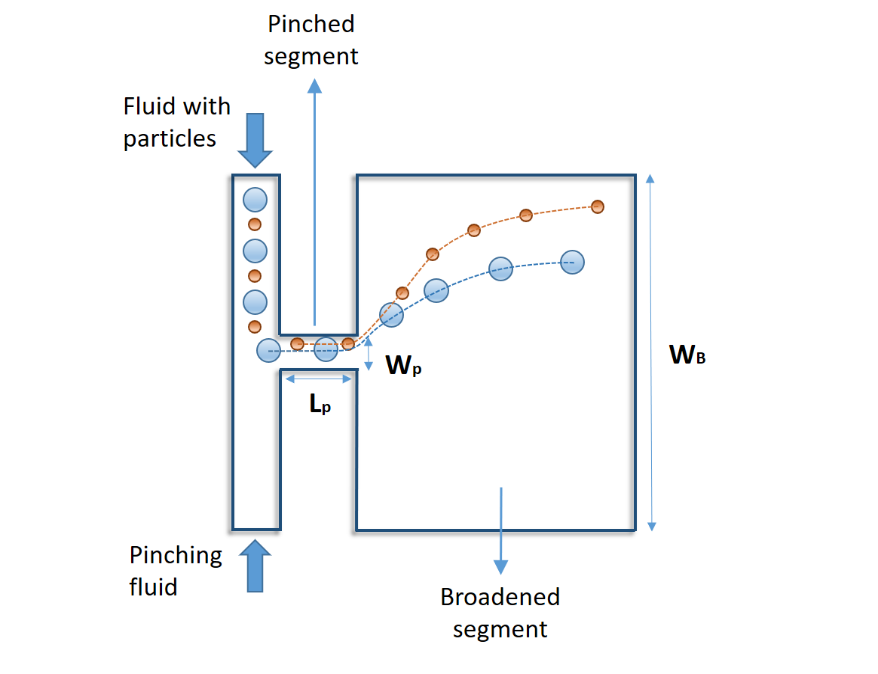


Figure S2: A demonstration for the PFF technique for separating particles of different sizes.

# Spectroscopic measurements setups

The setups used for the spectroscopic measurements using the Raman spectrometer, Raman microscope and FTIR microscope are shown in Figure S3.


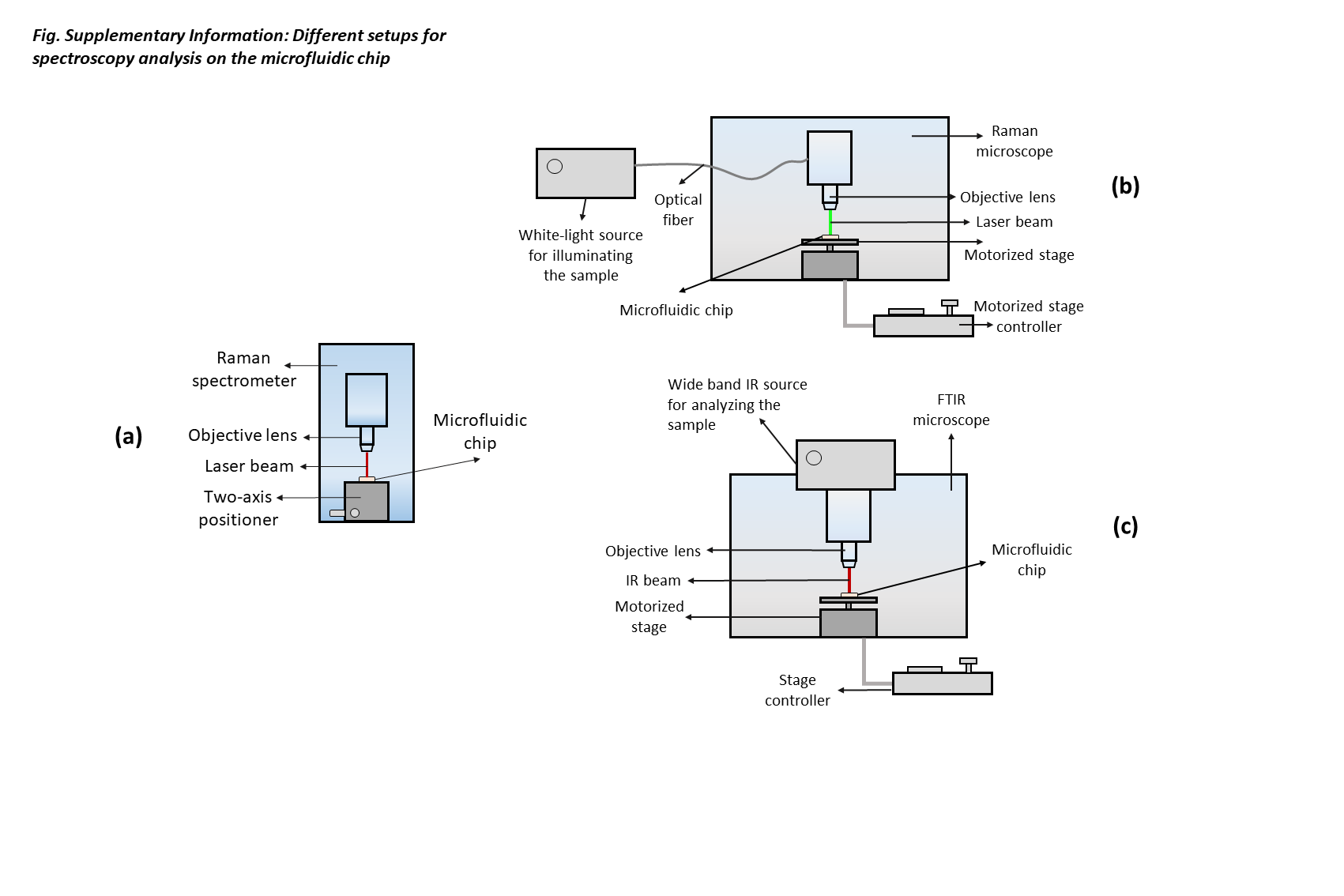


Figure S3: (a) Raman spectrometer setup (b) Raman microscope setup (c) FTIR microscope setup.

# Pre-concentration/filtration of water samples

To use the proposed chips with actual water samples these samples have to be pre-concentrated first to smaller volumes (in the order of few milliliters). This can be achieved with adequate speed using the setup demonstrated in Figure S4. The water sample containing the particles is poured from the top onto the filter, and with the help of the vacuum pump connected to the bottom container the sample filtering is achieved at a higher rate. After filtering the target sample volume, the filters are removed and the trapped particles can be re-suspended in a small volume of ultra-pure water to be inserted into the proposed chip.


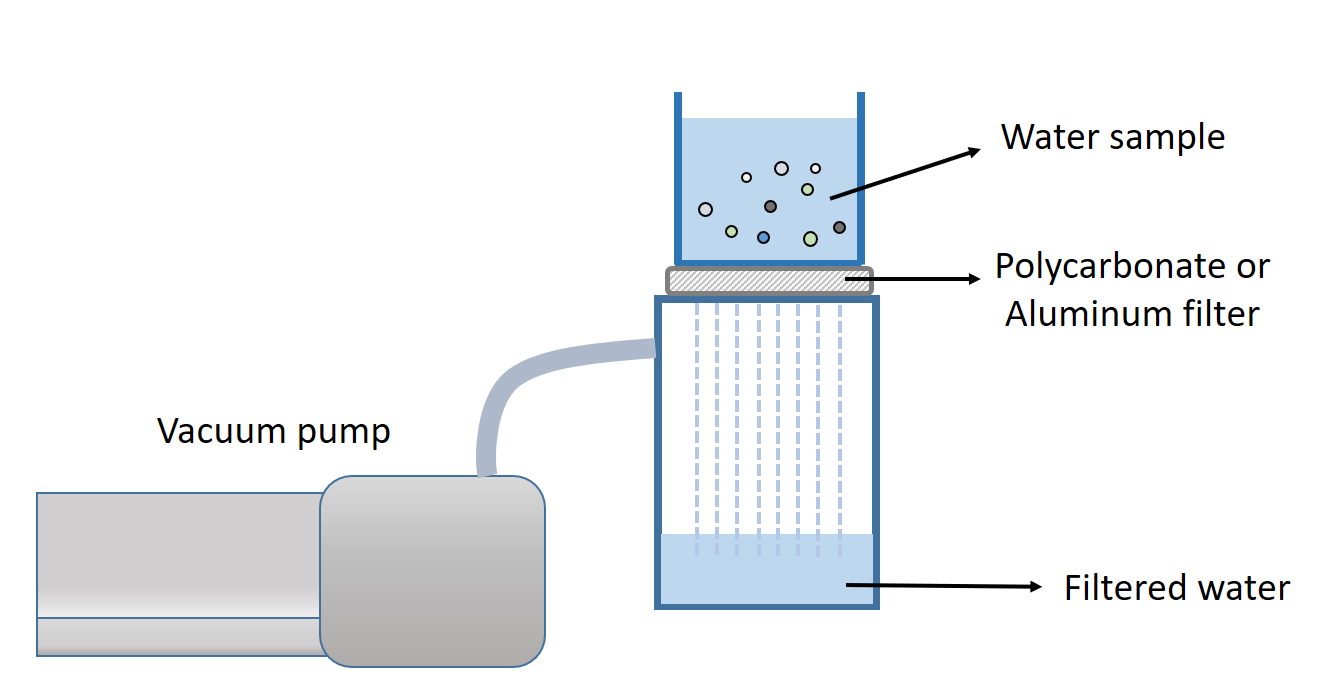


Figure S4: Setup used to filter water samples using polycarbonate or metallic filters with micro-scale pore size.

# Chip fabrication steps

The chips are fabricated using MEMS technology on Silicon wafers using Deep Reactive Ion Etching (DRIE) technique. The fabrication steps are listed below and demonstrated in Figure S5:

- Step 1: The Si wafer is oxidized, where the patterned oxide will be used later as a mask for etching Si to form the channels and different design features
- Step 2: Photoresist coating is made for the oxidized wafer
- Step 3: The photoresist is patterned using UV, and is removed in unwanted regions
- Step 4: The oxide is etched using the photoresist as a mask
- Step 5: The residual photoresist is removed
- Step 6: Si is etched using DRIE with the oxide acting as a mask
- Step 7: The residual oxide is removed
- Step 8: The wafer is flipped to start the back-etching to make the through-holes for fluid inputs and outputs
- Step 9: Photoresist coating for the back side of the wafer
- Step 10: Patterning of the photoresist is achieved
- Step 11: Etching of the oxide using the photoresist as a mask
- Step 12: Removal of residual photoresist
- Step 13: Etching of Si through the entire wafer thickness to make the through-holes
- Step 14: Removal of the residual oxide
- Step 15: Sealing the top side of the chip using glass-bonding or PDMS


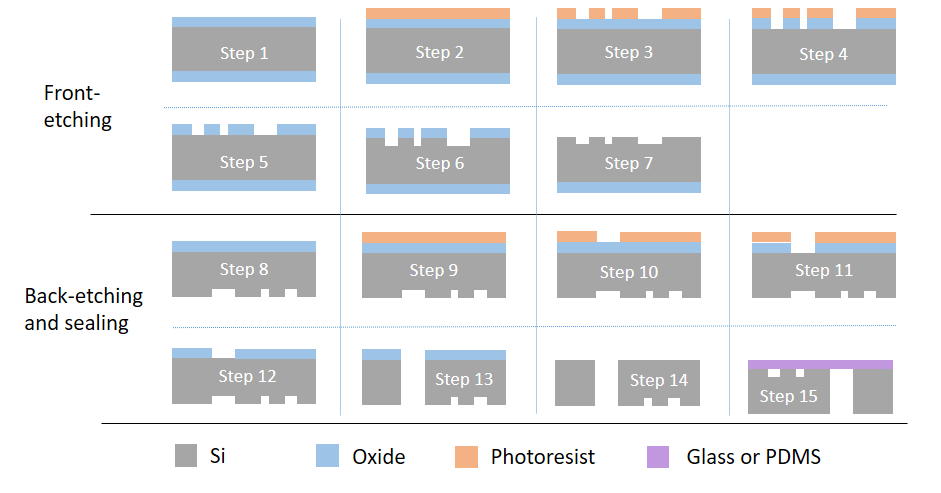


Figure S5: Fabrication steps of the proposed MEMS microfluidic chips.

# Supplementary References

S1. Kniggendorf, A. K., Wetzel, C. & Roth, B. Microplastics detection in streaming tap water with raman spectroscopy. *Sensors (Switzerland)* **19**, 12–14 (2019).

S2. Yamada, M., Nakashima, M. & Seki, M. Pinched flow fractionation: Continuous size separation of particles utilizing a laminar flow profile in a pinched microchannel. *Anal. Chem.* **76**, 5465–5471 (2004).
